# A Peptide-Based PD1 Antagonist Enhances T-Cell Priming and Efficacy of a Prophylactic Malaria Vaccine and Promotes Survival in a Lethal Malaria Model

**DOI:** 10.3389/fimmu.2020.01377

**Published:** 2020-07-09

**Authors:** Timothy W. Phares, Vinayaka Kotraiah, Deshapriya S. Karunarathne, Jing Huang, Cecille D. Browne, Peter Buontempo, Marc Mansour, Amy R. Noe, Michelle N. Wykes, James Pannucci, Moriya Tsuji, Gabriel M. Gutierrez

**Affiliations:** ^1^Explorations in Global Health (ExGloH), Leidos Inc., Frederick, MD, United States; ^2^QIMR Berghofer, Brisbane, QLD, Australia; ^3^The Aaron Diamond AIDS Research Center, New York, NY, United States; ^4^Leidos Life Sciences, Leidos Inc., Frederick, MD, United States

**Keywords:** T-cell, vaccine, malaria, peptide, therapeutic, programmed cell death-1, sporozoite

## Abstract

The blockade of programmed cell death-1 (PD1) and its ligand PDL1 has been proven to be a successful immunotherapy against several cancers. Similar to cancer, PD1 contributes to the establishment of several chronic infectious diseases, including malaria. While monoclonal antibodies (mAbs) targeting checkpoint receptors are revolutionary in cancer treatment, the immune-related adverse events (irAEs) may prevent their utilization in prophylactic and therapeutic treatments of infectious diseases. The irAEs are, in part, due to the prolonged half-life of mAbs resulting in prolonged activation of the immune system. As an alternative modality to mAbs, peptides represent a viable option because they possess a shorter pharmacokinetic half-life and offer more formulation and delivery options. Here, we report on a 22-amino acid immunomodulatory peptide, LD01, derived from a *Bacillus* bacteria. When combined prophylactically with an adenovirus-based or irradiated sporozoite-based malaria vaccine, LD01 significantly enhanced antigen-specific CD8^+^ T cell expansion. Therapeutically, LD01 treatment of mice infected with a lethal malaria strain resulted in survival that was associated with lower numbers of FOXP3^+^Tbet^+^CD4^+^ regulatory T cells. Taken together, our results demonstrate that LD01 is a potent immunomodulator that acts upon the adaptive immune system to stimulate T cell responses both prophylactically and therapeutically.

## Introduction

Despite decades of research, the world is still without a malaria vaccine capable of establishing a long-lasting anamnestic response. The parasite's resistance to traditional vaccinology suggests that the pathogen is actively employing immune suppression mechanisms. Indeed, even after years of exposure to high *P. falciparum* transmission, there is no indication of acquired, sterile immunity to *P. falciparum* infections, while clinical immunity to blood-stage malaria can be achieved (1). Thus, adults in high-transmission areas often experience asymptomatic infections and remain reservoirs for parasite transmission via mosquitos. This is in contrast to many viral and bacterial pathogens, which generally induce life-long immunity after a single exposure (2).

To minimize damage to the host from uncontrolled inflammation during infection, the immune system is tightly controlled by several soluble as well as contact-dependent mechanisms that limit activation and immune-mediated pathology. The programmed death 1 (PD1) receptor is a well-understood checkpoint protein that negatively regulates immune responses. Checkpoint receptors have been implicated in establishing immune exhaustion, not unlike in cancer, during parasitic infection, thereby allowing the parasite to evade immunity (3). Indeed, continuous exposure to *P. falciparum* drives the expansion of atypical memory B cells and increased frequencies of CD4^+^ T cells expressing phenotypic markers of exhaustion including PD1 (4–6). Additionally, in mice with *Plasmodium* infection, the blockade of PD1/PDL1, as well as lymphocyte activation gene 3 (LAG-3)/MHC II interactions, restores T cell function, culminating in the rapid clearance of blood-stage parasites (7). Furthermore, PD1-deficient mice rapidly clear the parasites, unlike infections in wild-type mice (3, 6), supporting PD1-mediated suppression of anti-malarial immunity.

The most advanced malaria vaccine identified to date is the RTS,S/AS01E, which has shown a limited efficacy of 43.6% in the first year of administration that decreases to 16.8% by the fourth year (8). The primary mechanism of protection by the RTS,S vaccine is reported to be largely based on humoral responses (9). Another leading malaria vaccine approach involves the administration of radiation-attenuated sporozoites (RAS) (10). In this approach, CD8^+^ T cell responses were shown to be responsible for long-term protection following RAS immunization (11–16), with RAS-induced antibodies playing a minimal role (17). High numbers of circulating memory CD8^+^ T cells have been shown to correlate with the maintenance of protection against infection (18). Further, a population of RAS-induced resident memory T cells is essential for protection against malaria sporozoite challenge (19–23). Several groups have also identified a unique mechanism of CD8^+^ T cell elimination of parasite-infected hepatocytes in which T cells “cluster” infected cells (24–28). Thus, it has become apparent that T cells are key players in immune responses to *P. falciparum* and that the development of a T cell modulator would have the potential to enhance responses particularly to liver-stage malaria vaccines.

It is clear that overcoming the suppression of the adaptive immune responses in the context of natural, endemic infections is crucial for a practical vaccine to be administered in a malaria-endemic region. It is also apparent that the most effective vaccines currently require adaptive immunity for protection. Therefore, unlike traditional adjuvants, immune modulators that inhibit checkpoint receptors can have a distinctly different mechanism by which they trigger a direct expansion of vaccine antigen-specific CD4^+^ and CD8^+^ T cells. However, the potential side effects associated with the current mAb-based immune checkpoint cancer treatments, as well as the potential exacerbation of cerebral malaria, have impeded attempts to progress checkpoint inhibitors for use in malaria (29). Nevertheless, with the spread of drug resistance, the absence of a truly efficacious vaccine, and the promise of alternatives such as soluble PD-L2 that can generate long-term protection with a reduced incidence of cerebral malaria (30), new modalities that can be safely adopted for infectious disease vaccines and therapeutics require investigation.

Accordingly, we undertook a strategy of discovering and developing peptide-based checkpoint inhibitors as an alternative modality to mAbs. We opted for this approach because peptide-based checkpoint inhibitors provide a shorter pharmacokinetic profile. In the current study, we identified LD01, a 22-amino acid pharmacophore from the Cry1A toxin of *Bacillus thuringiensis*. We derived LD01 from the sequence motif similarity to a PD1 peptide antagonist that we had previously identified by screening a random peptide library (31). Unlike our previously described PD1 peptide-based antagonists (31), LD01 has been predicted via computational modeling to bind a hydrophobic groove on the PD1 receptor where its predicted binding site overlaps only marginally with that of PDL1; however, LD01 antagonizes PD1 in a cell-based reporter assay. Moreover, LD01 significantly enhanced Plasmodium-specific CD8^+^ T cell expansion in mice, when administered with an adenovirus-based or irradiated sporozoite-based malaria vaccine. Therapeutically, LD01 treatment promoted the survival of mice infected with a lethal Plasmodium strain that was associated with a lower number of FOXB^+^Tbet^+^CD4^+^ regulatory T cells. Collectively, these data demonstrate that LD01 modulates T cell responses and has the potential to enhance prophylactic as well as therapeutic measures.

## Materials and Methods

### PD1:PDL1 Cell-Based Reporter Assay

For the PathHunter® PD-1 signaling assay (Catalog number 93-1104C19; Eurofins DiscoverX; Fremont, CA), Jurkat cells expressing PD1 and SHP1 proteins, each fused to a fragment of DiscoverX's enzyme fragment complementation (EFC) system, were co-incubated with ligand-presenting cells. This resulted in PD1 activation and SHP1 recruitment to the PD1 receptors, bringing together the two EFC fragments and generating a light signal. In this assay, LD01, LD12 (20 and 100 μM), and anti-PD1 mAb controls were assessed at 10 different concentrations. In brief, PD-1 expressing Jurkat cells (20,000 cells per well) were seeded in a total volume of 50 μL into white-walled, 96-well microplates in assay buffer. Serial dilution of LD01 stock was performed to generate an 11X sample in assay buffer. Ten microliters of the 11X test sample were added to PD1 cells and incubated at 37°C for 60 min. U2OS cells expressing PD-L1 (50 μL, 30,000 cells per well in assay buffer) were then added to the assay. Cells in co-culture were incubated at room temperature (RT) for 2 h (PD1 assay). The assay signal was generated using the PathHunter Bioassay Detection kit for both assays. Detection reagent 1 (10 μL) was added to the assay and incubated at RT for 15 min. Detection reagent 2 (40 μL) was added to the assay and incubated at RT for 60 min. Microplates were read following signal generation with a PerkinElmer Envision^TM^ instrument (PerkinElmer, Waltham, MA) for chemiluminescent signal detection. LD01 activity and anti-PD1 mAb activities were analyzed using the CBIS data analysis suite (ChemInnovation, San Diego, CA). For antagonist mode assays, percentage inhibition for the peptides was calculated using the following formula: percent inhibition efficacy = 100% × [1 – (mean RLU of test sample – mean RLU of vehicle control) / (mean RLU of EC80 control – mean RLU of vehicle control)].

### Mice for AdPyCS Vaccine and RAS Vaccine Studies

Six to eight-week-old female BALB/c mice were purchased from The Jackson Laboratory (Bar Harbor, ME). All mice were maintained under standard conditions in the Laboratory Animal Research Center of The Rockefeller University and the protocol was approved by the Institutional Animal Care and Use Committee at The Rockefeller University (Assurance No. A3081-01).

### AdPyCS Vaccine, *P. yoelii* Parasites, and RAS Vaccine

A recombinant serotype 5 adenovirus that expressed *P. yoelii* circumsporozoite protein (PyCS), AdPyCS, was constructed, as previously described (32). A wild-type non-lethal rodent malaria parasite strain, *P. yoelii* 17XNL, was maintained in the insectary facility of the Division of Parasitology, Department of Microbiology at New York University School of Medicine. *P. yoelii* sporozoites were obtained from dissected salivary glands of infected *Anopheles stephensi* mosquitoes 2 weeks after an infective blood meal (32). For the RAS vaccine, *P. yoelii* sporozoites were radiation-attenuated by exposure to 12,000 rad (33).

### ELISpot Assay to Measure Antigen-Specific CD8 T Cells

The relative numbers of splenic PyCS-specific, IFN-γ-secreting CD8^+^ T cells of AdPyCS- or RAS-immunized mice were determined by an ELISpot assay, using a mouse IFN-γ ELISpot kit (Abcam, Cambridge, MA) and a synthetic 9-mer peptide, SYVPSAEQI (Peptide 2.0 Inc., Chantilly, VA) corresponding to the immunodominant CD8^+^ T cell epitope within PyCS, as previously described (32). Briefly, after the collection of splenocytes from mice 12 days after AdPyCS or RAS immunization, 5 × 10^5^ splenocytes were placed on each well of the 96-well ELISpot plates pre-coated with IFN-γ antibody and incubated with the peptide at 5 μg/mL for 24 h at 37°C in a CO_2_ incubator. After the ELISpot plates were washed, they were incubated with biotinylated anti-mouse IFN-γ antibody for 2–3 h at RT, followed by incubation with avidin-conjugated with horseradish peroxidase for 45 min at RT in the dark. Finally, the spots were developed after the addition of the ELISpot substrate (Abcam). To identify the number of IFN-γ-secreting CD8^+^ T cells in each well, the mean number of spots (for duplicates) counted in the wells incubated with splenocytes in the presence of the peptide was subtracted by the mean number of spots (for duplicates) counted in the wells that were incubated with splenocytes only.

### *P. yoelii* 17 XNL Sporozoite Challenge and Monitoring of Parasitemia

Sporozoite challenge experiments were performed, as described previously (32). Briefly, immunized mice were administered 100 live *P. yoelii* 17 XNL sporozoites IV via the tail vein. Parasitemia was monitored from days 3 to 10 after sporozoite challenge by detecting the presence of parasitized red blood cells in thin blood smears to assess complete protection against malaria. Briefly, a drop of blood was collected from the mouse tail vein for thin blood smears on pre-cleaned glass slides. Thin blood smears were fixed with absolute methanol and then stained with diluted Giemsa stain (1:20, v/v) (Sigma-Aldrich, St. Louis, MO) for 10 min. The presence of parasitemia (parasitized red blood cells) was examined using a 100× oil immersion objective under a microscope. For each blood smear ten distinct fields were examined.

### Mice for *P. yoelii* YM Studies

Specific pathogen-free C57BL/6J wild-type female mice, 8–12 weeks of age, were obtained from the Animal Resources Centre (Perth, Australia). Mice were housed in the QIMR animal research facility. All procedures were approved and monitored by the QIMR Animal Ethics Committee (Approval number A0209-622M) in accordance with the “Australian code of practice for the care and use of animals for scientific purposes” (NHMRC, Australian). The sample size was estimated based on previous studies with similar assays, using the same parasites. For experiments with multiple groups, all mice were first infected and then randomly assigned into treatment groups. No blinding was undertaken.

### *P. yoelii* YM Infection and Monitoring

Cohorts of 3–10 wild-type mice were infected IV with 10^4^ *P. yoelii* YM parasitized red blood cells (pRBCs) freshly obtained from previously infected C57BL/6J mice. Tail-tip blood films were made every 1–2 days, stained using the Quick Dip modified Wright-Giemsa stain (ThermoFisher Scientific) and examined for parasitemia, for up to 60 days. The percentage of pRBCs was assessed by counting at least 300 RBCs during parasitemia >1% and 20 fields with ~10,000 cells at other times. The mean percentage parasitemia, shown in several figures, is the mean percentage pRBC of total RBC, from individual mice in a group. Mice were monitored using the following clinical assessment criteria for distress during the period of the experiment (Table 1). If any of the assessment criteria reached Grade 2, the mouse was euthanized. If each of the criteria reached a Grade 1 (cumulative score equal to 4), and this situation persisted for over 120 h, the mouse was euthanized.

**Table 1:** Assessment criteria.

**Criteria**	**Grade 0**	**Grade 1**	**Grade 2**
Weight loss	<10%	>10 to <20%	>20%
Posture	Normal	Mild to moderate hunching	Severe hunching impairs movement
Activity	Normal	Mild to moderately decreased activity	Stationary unless stimulated
Fur texture	Normal	Ruffling	N/A

### Treatment of *P. yoelii* YM-Infected Mice

Groups of 3–10 mice were infected with *P. yoelii* YM pRBC and treated twice daily with 100–200 μg/injection/mouse of LD01. Parallel groups of mice were treated with a single dose of 200 μg of control rat Ig or anti-PD1 antibody. For protection studies, mice were monitored daily, as described above. For cellular studies, at day 6, spleens were taken from infected mice and processed individually. Spleens from naïve mice were used as controls. Spleens were digested with collagenase to release DCs and maximal numbers of other cell types. T cell subset markers included (CD3, CD4, and CD8) for TH1 (Tbet), Treg (FOXP), and checkpoint markers (PD1).

### Data Analysis

Statistical analyses were performed using GraphPad Prism (GraphPad Software, Inc., La Jolla, CA). One-way analysis of variance (ANOVA) followed by Dunnett's test was used to determine the differences between three or more groups, whereas an unpaired *t*-test and Mann-Whitney test were used if the comparison was performed between two groups. Correlative analysis was assessed using the Mantel-Cox test.

## Results

### PD1 Receptor Signaling Is Impaired by LD01

**Figure 1:**
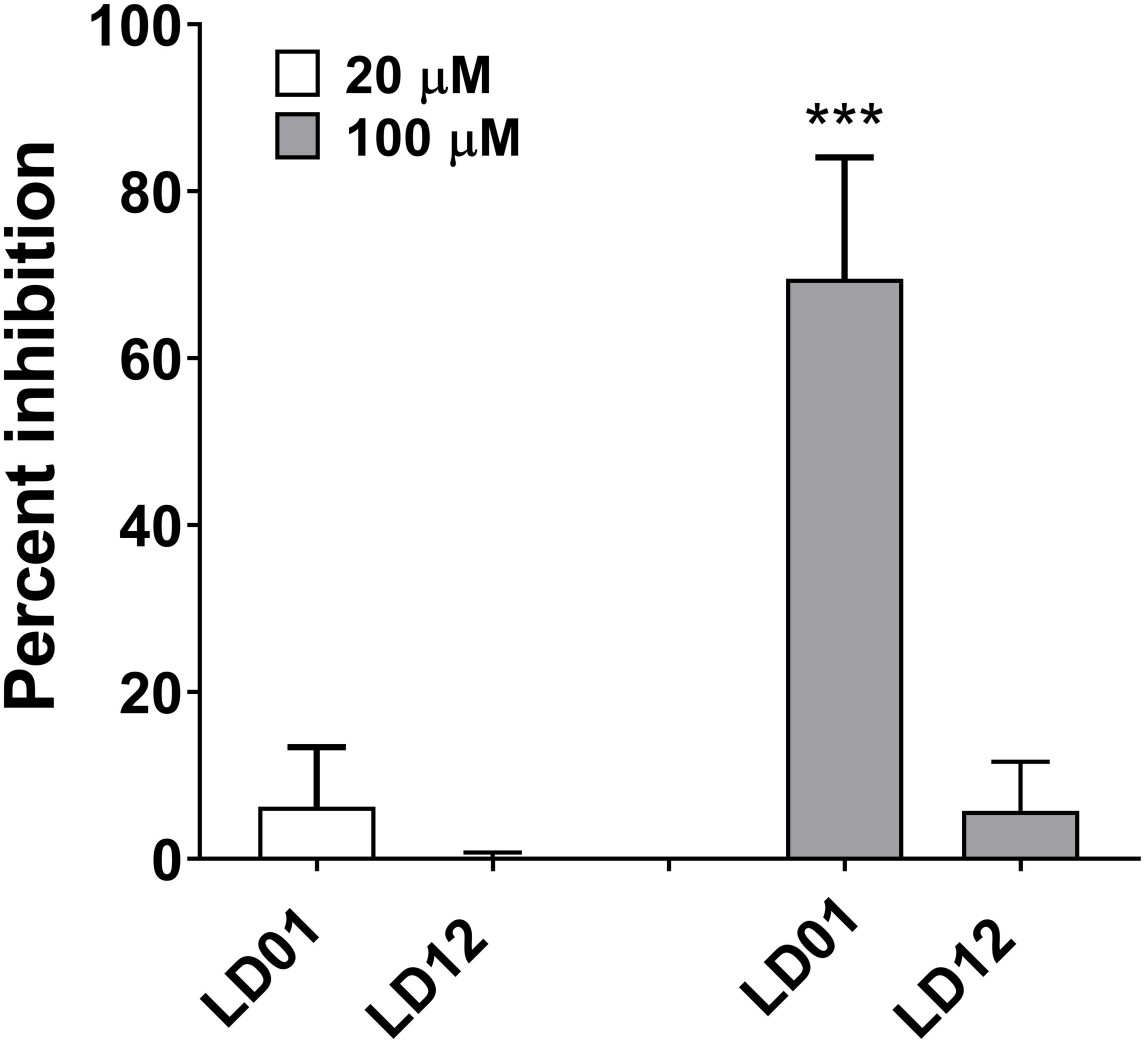
LD01 reduces PD1 receptor signaling in a functional cell-based assay. Jurkat cells expressing PD1 were incubated with LD01 or LD12 at 20 μM and 100 μM for 60 min. PDL1 expressing cells were then added to the assay and co-cultured for 2 h. Chemiluminescent signal was detected, and percent inhibition for the peptides was calculated using the formula described in the section Materials and Methods. In this assay PD1 receptor signaling refers to SHP1 recruitment to the PD1 receptor where Jurkat cells expressing PD1 and SHP1 proteins, each fused to a fragment of an EFC system, were co-incubated with ligand-presenting cells. This results in PD1 activation and SHP1 recruitment to the PD1 receptors, bringing together the two EFC fragments and generating a light signal. Data represent four independent experiments and are expressed as the mean ± SD percent inhibition. Significant differences between LD01 and LD12 at 100 μM were determined using a two-tailed Unpaired *t*-test and are denoted by ***p < 0.0005.

To demonstrate the ability of LD01 to interfere with PD1:PDL1 interaction, the PathHunter PD1 Signaling Bioassay (Eurofins DiscoverX) was performed. In this assay, Jurkat cells expressing PD1 and SHP1 proteins, each fused to an enzyme fragment complementation system, are co-incubated with PD1 ligand-presenting cells. This results in PD1 activation and SHP1 recruitment to PD1, bringing together the two EFC fragments and generating a light signal. The addition of an anti-PD1 inhibitor, such as an antibody, blocks the PD1:PDL1 interaction resulting in a decreased chemiluminescent signal. Of note, the IC_50_ of an anti-PD1 antibody run in parallel with the peptides was ~80 nM (data not shown). LD01 was tested at two concentrations, 20 and 100 μM. In addition to LD01, we tested LD12, a derivative of LD01 that has a single amino acid change. While incubation of 20 μM LD01 showed minimal inhibition of PD1 signaling, the treatment of cells with 100 μM demonstrated a mean inhibition of ~70% (Figure 1). By contrast, LD12 at 100 μM showed a mean inhibition of ~6%, indicating that the single amino change significantly reduced its activity in this cell-based assay. These results indicate that LD01 impairs receptor signaling through inhibition of the PD1:PDL1interaction.

### LD01 Increases Antigen-Specific CD8^+^ T Cell Expansion Following Adenovirus-Based Vaccination

**Figure 2:**
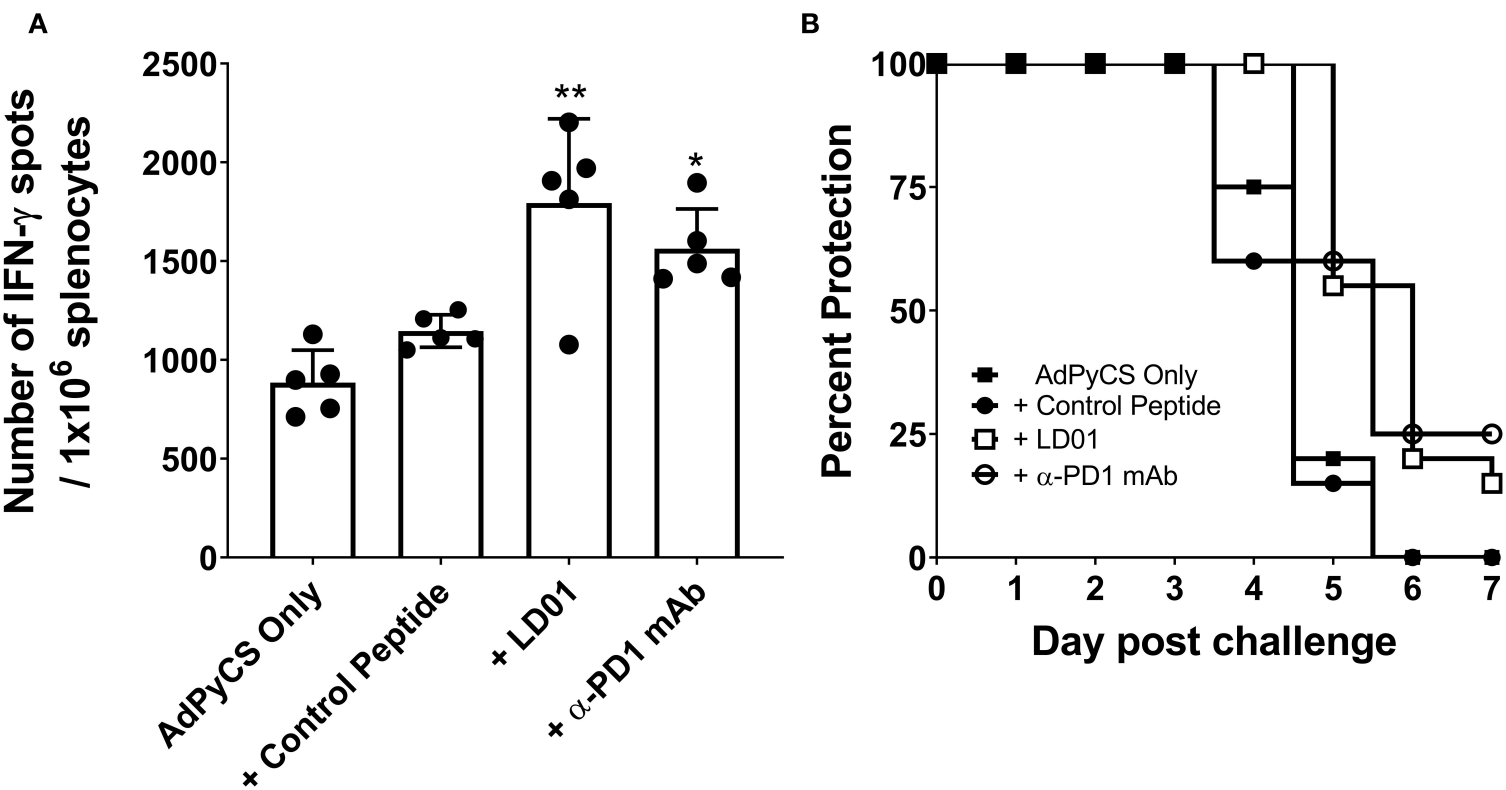
LD01 increases antigen-specific CD8^+^ T cell numbers following AdPyCS vaccination. **(A)** At day 12 post-AdPyCS immunization, immunogenicity was assessed by measuring the number of splenic PyCS-specific, IFN-γ-secreting CD8^+^ T cells using the ELISpot assay after stimulation with the H-2kd restricted CD8 epitope SYVPSAEQI. Data are expressed as the mean ± SD. Data from one of two independent experiments are shown. Significant differences between AdPyCS + control peptide and treated mice were determined using a one-way ANOVA multiple comparisons test and are denoted by *p < 0.05 and **p < 0.005. **(B)** Immunized and treated mice were challenged with Py 17XNL sporozoites IV. Data represent two independent experiments (*n* = 10/group/experiment) and are expressed as the mean percent protection. Parasitemia was assessed beginning at day 3 post-challenge, and sterile protection was defined as the absence of parasite detection in the blood through day 7 by microscopic examination of Giemsa-stained thin smears.

PD1 blockade has been shown to increase the expansion of CD8^+^ T cells by inhibiting PD1 regulation of T cell differentiation (34). To evaluate whether LD01 alters antigen-specific CD8^+^ T cell expansion following vaccination, we used the recombinant replication-defective adenovirus serotype 5 expressing the entire *P. yoelii* circumsporozoite protein (AdPyCS) as a model vaccine (32). The PyCS protein possesses an immunodominant H-2K^d^-restricted CD8^+^ T cell epitope, SYVPSAEQI, which is known to be protective (32). AdPyCS was injected intramuscularly (IM) into mice in the hind limb. Of note, the number of splenic PD1^+^ CD8 T cells at 4 days post-vaccination increases by ~35% relative to naïve controls (31). Following AdPyCS injection, mice were treated intraperitoneally (IP) with a control peptide, LD01, or α-PD1 mAb (Figure 2A). The control peptide was OVA_323−339_. The dose of peptides and α-PD1 mAb delivered in this model was 200 μg/injection/mouse, administered on days 1, 3, 5, and 7. Spleens were harvested on day 12, and the relative number of splenic PyCS-specific, IFN-γ-secreting CD8^+^ T cells was assessed using an ELISpot assay. LD01 treatment significantly enhanced the number of PyCS-specific, IFN-γ-secreting CD8^+^ T cells by ~2-fold relative to AdPyCS control immunization (Figure 2A). This increase was comparable to that of the α-PD1 mAb-treated group, which demonstrated a ~1.8-fold change in the number of PyCS-specific, IFN-γ-secreting CD8^+^ T cells. Taken together, the data support the notion that LD01 treatment results in enhanced expansion of vaccine antigen-specific T cells *in vivo*.

Enhanced antigen-specific CD8^+^ T cell expansion may result in increased vaccine efficacy. Thus, parasite challenge studies were conducted in the presence of LD01. For these studies, mice were immunized IM with a suboptimal dose (10^9^ virus particles) of AdPyCS, which resulted in no protection following parasite challenge (Figure 2B). At days 1, 3, 5, and 7 post-immunization, mice were treated IP with 200 μg of control peptide, LD01, or α-PD1 mAb. Twelve days post-immunization, mice were challenged IV with 100 live *P. yoelii* sporozoites. Parasitemia (parasitized red blood cells) was assessed via blood smears beginning at day 3 post-challenge, with the absence of parasitemia through day 7 post-infection representing sterile immunity. As shown in Figure 2B, no sterile protection was seen in mice immunized with AdPyCS alone. However, the treatment of AdPyCS-immunized mice with LD01 or α-PD1 mAb resulted in 15% and 25% protection, respectively (Figure 2B). While the changes in sterile protection were not significant, the trends in enhanced protection argue that modulation of PD1 activity can alter the efficacy of a sub-optimally dosed vaccine. Further, when the time of patency of the AdPyCS alone mice (4.8 ± 0.6 days) was compared to LD01 (5.5 ± 0.6 days) or α-PD1 mAb (5.5 ± 0.5 days) treated mice, significant differences were detected using a two-tailed, Unpaired *t*-test, p = 0.002 and 0.003, respectively. There was not a significant difference in time of patency between the AdPyCS alone (4.8 ± 0.6 days) and control peptide (4.7 ± 0.7 days).

### LD01 Enhances Antigen-Specific CD8^+^ T Cell Expansion Following RAS-based Vaccination

**Figure 3:**
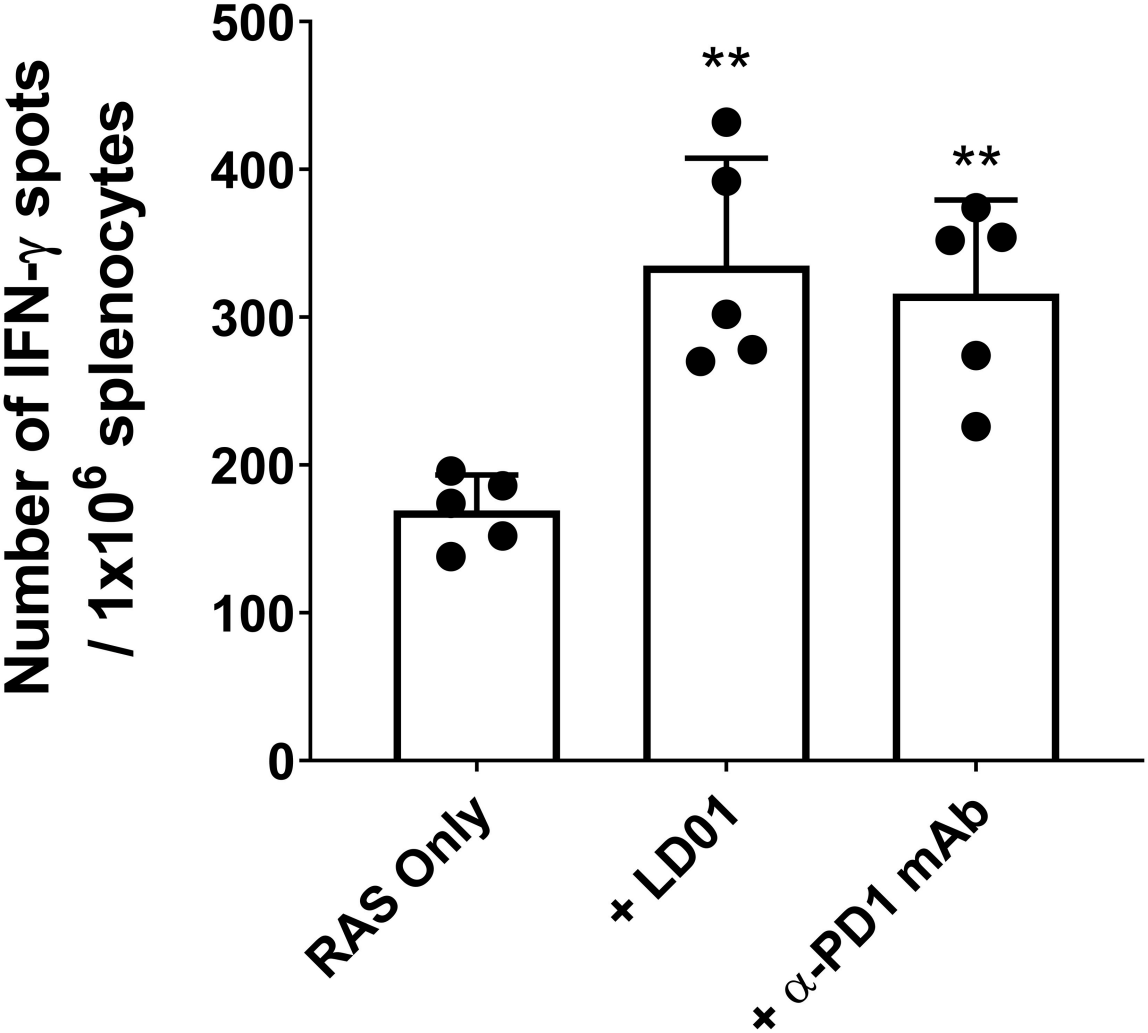
LD01 enhances antigen-specific CD8^+^ T cell numbers following RAS vaccination. At day 12 post-RAS immunization, immunogenicity was assessed by measuring the number of splenic PyCS-specific, IFN-γ-secreting CD8^+^ T cells using the ELISpot assay after stimulation with the H-2kd restricted CD8 epitope SYVPSAEQI. Data are expressed as the mean ± SD. Data from one of two independent experiments are shown. Significant differences between RAS alone and treated mice were determined using a two-tailed Unpaired *t*-test and are denoted by **p < 0.005.

To confirm the effect of LD01 on CD8^+^ T cell expansion, we also evaluated the effect of LD01 treatment on the induction of antigen-specific CD8^+^ T cells following immunization with a RAS-based malaria vaccine. For these studies, mice were immunized IM with 1 × 10^5^ RAS in their hind limb and then immediately treated with a single IP injection of 20 μg LD01 or 100 μg α-PD1 mAb (Figure 3). Note that the dose and number of treatments differ from those used with AdPyCS (Figure 2A). Twelve days following RAS immunization, spleens were harvested, and the relative number of splenic PyCS-specific, IFN-γ-secreting CD8^+^ T cells was assessed by ELISpot assay. As shown in Figure 3, a single dose of LD01 significantly enhanced the number of PyCS-specific, IFN-γ-secreting CD8^+^ T cells relative to that induced by RAS alone with a ~2-fold increase. This increase was comparable to that observed with α-PD1 mAb. These data corroborate the results from Figure 2A demonstrating that LD01 enhances the expansion of vaccine-induced specific CD8^+^ T cells. Moreover, the data show that a single dose of LD01 at the time of vaccination is efficacious. Taken together, the adenovirus-based and RAS-based vaccine results suggest that the effect of LD01 on antigen-specific CD8^+^ T cell expansion is vaccine-platform-independent.

### LD01 Promotes Survival in Lethal Malaria

**Figure 4:**
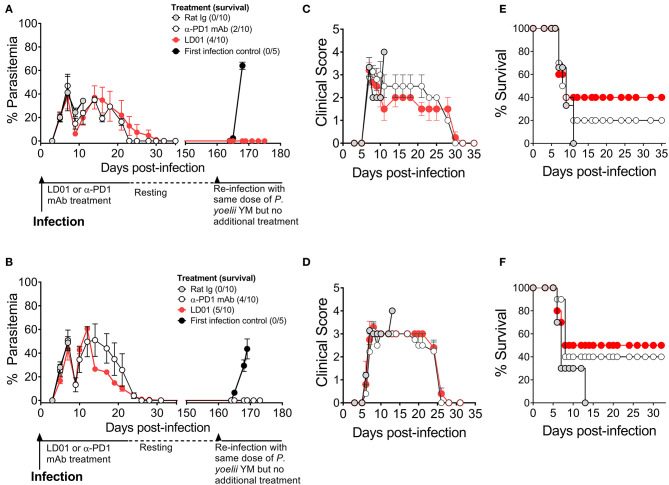
LD01 improves survival and promotes a long-lived protection memory response from lethal malaria. **(A–D)** 10^4^ Py YM-infected red blood cells were injected IV into mice (*n* = 10), treated IP with LD01 or antibody, and monitored daily for **(A,B)** parasitemia, **(C,D)** clinical scores and **(E,F)** survival. The dose of LD01 delivered was 100 or 200 μg/injection/mouse administered twice daily on either days 1, 2, 3, 4, and 5 **(A)** or days 3, 4, 5, 6, and 7 **(B)**. 200 μg doses were given on days 1 and 2 **(A)** or days 3 and 4 **(B)** with 100 μg doses administered on days 3, 4, and 5 **(A)** or days 5, 6, and 7 **(B)** respectively. The dose of anti-PD1 mAb and control rat IgG delivered was 200 μg/injection/mouse administered daily on either days 1, 3, and 5 **(A)** or days 3, 5, and 7 **(B)**. **(A,B)** Parasitemia was assessed beginning at day 3 and blood films were made every 1–2 days up to day 35. Surviving mice were then rested. On day 160, along with a new group of first infection control mice (*n* = 5), they were infected with 10^4^ Py YM-infected red blood cells **(A,B)**. The mice were monitored daily for survival and parasitemia. The two surviving mice treated with α-PD1 mAb in **(A)** were not re-challenged. Data are expressed as the mean ± SEM percent of malaria-positive RBC of total RBC **(A)** or ± SEM of clinical scores **(B)**.

PD1 is implicated in human malaria and shown to mediate the lethality of the *P. yoelii* (Py) YM strain (35). Thus, to test the protective capacity of LD01 against a lethal blood-stage malarial infection, we infected mice with a lethal Py YM strain of malaria and treated them with LD01, α-PD1 mAb, or control rat Ig. All mice were injected IV with Py YM-infected red blood cells and then treated IP. The dose of LD01 delivered was 100 or 200 μg/injection/mouse administered twice daily on either days 1, 2, 3, 4, and 5 (Figure 4A) or days 3, 4, 5, 6, and 7 (Figure 4B). 200 μg doses were given on days 1 and 2 (Figure 4A) or days 3 and 4 (Figure 4B) with 100 μg doses administered on days 3, 4, and 5 (Figure 4A) or days 5, 6, and 7 (Figure 4B), respectively. The dose of α-PD1 mAb and control rat Ig delivered was 200 μg/injection/mouse administered daily on either days 1, 3, and 5 (Figure 4A) or days 3, 5, and 7 (Figure 4B). In both studies, all mice infected with Py YM and treated with control rat Ig had to be euthanized within 13 days due to high parasitemia and/or clinical symptoms as described in the Methods (Figures 4A–F). By contrast, 40 to 50% of Py YM-infected mice treated with LD01 survived (Figures 4E,F) and cleared the infection in 35 days (Figures 4A,B) with clinical symptoms subsiding (Figures 4C,D). Similarly, 20 to 40% of the mice treated with α-PD1 mAb survived (Figures 4E,F) and cleared the infection (Figures 4A,B). Notably, the increased survival for the LD01 and α-PD1 mAb treatments was significant (*p* = 0.04 and *p* = 0.003, respectively, using a Mantel-Cox test) when the two independent studies were combined. To address whether surviving mice developed a long-lived protective memory response, surviving mice were re-infected with Py YM on day 160 (Figures 4A,B). In parallel, a naïve cohort of mice was infected to confirm the lethality of the parasite. Note that the two surviving mice treated with α-PD1 mAb in Figure 4A were not re-challenged. As anticipated, all naïve mice succumbed or had to be euthanized within 10 days due to increasing parasitemia and/or clinical symptoms. Most significantly, 100% of the LD01 and α-PD1 mAb-treated mice survived the re-infection and controlled parasitemia (Figures 4A,B). Notably, low parasitemia was detected upon re-infection with peak levels reaching 0.006 ± 0.01% (Figure 4A) and 0.123 ± 0.267% (Figure 4B) for the LD01 cohorts and 0.101 ± 0.193% for the α-PD1 mAb cohort (Figure 4B). Taken together, these data show that LD01 promotes survival in a lethal malaria model and induces a protective memory response.

### LD01-Mediated Survival Is Associated With Fewer FOXP3^+^Tbet^+^ CD4 T Cells

**Figure 5:**
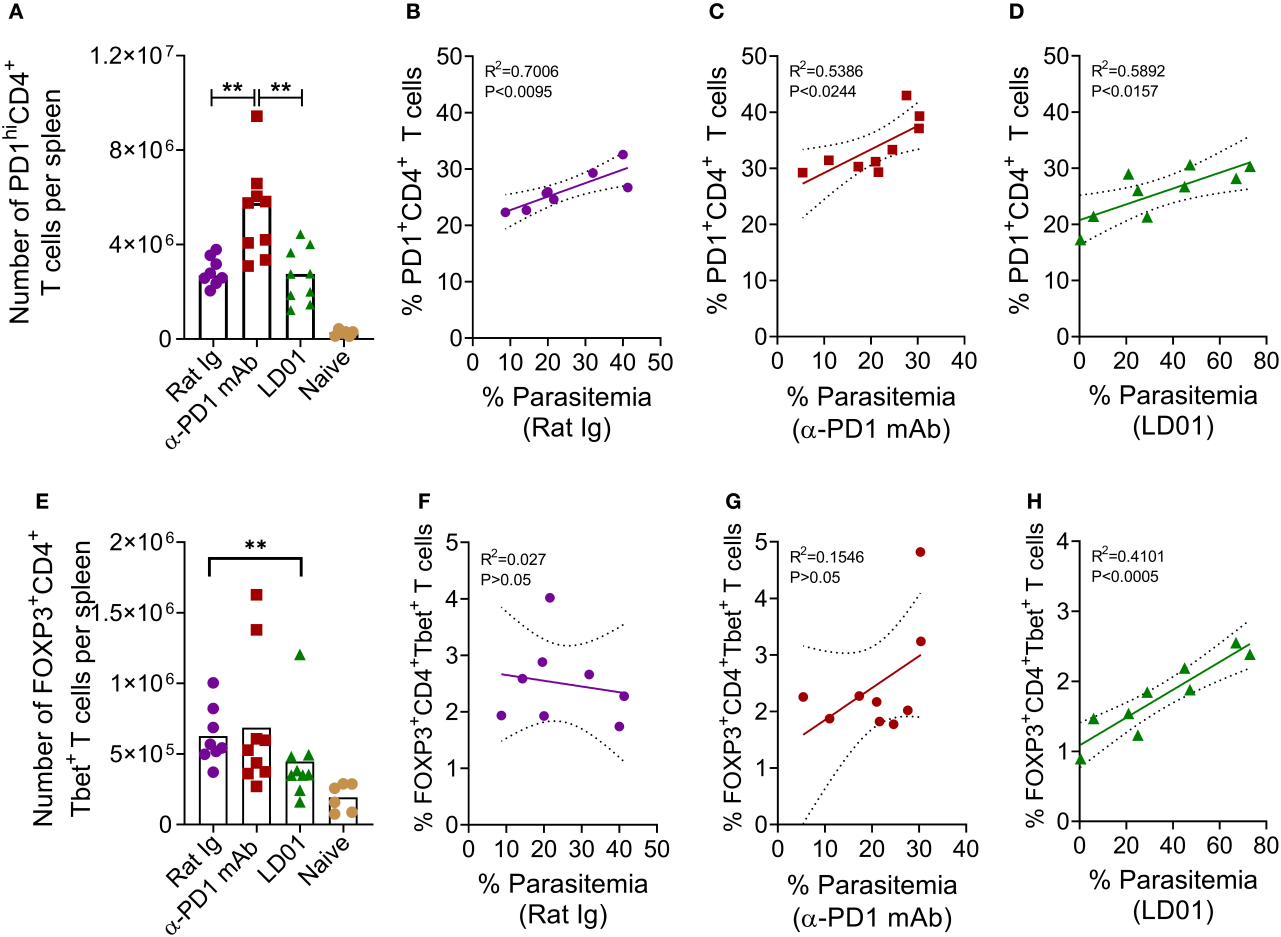
LD01 decreases the number of splenic FOXP3^+^Tbet^+^ CD4 T cells. 10^4^ Py YM-infected red blood cells were injected IV into mice and treated IP with LD01 or antibody. Spleens from individual mice were harvested at day 6 post-infection, stained for CD3, CD4, CD8, Tbet, FOXP3, and PD1, and analyzed by flow cytometry. Spleens from naïve mice were used as controls. The numbers of PD1^hi^CD4^+^ T cells and FOXP3^+^Tbet^+^ CD4 T cells per spleen are shown in **(A,E)**. Pooled data depict the mean of two independent experiments with n ≥ 3 mice per experiment. Significant differences were determined by Mann–Whitney test and are denoted by **p < 0.01. Parasitemia in each mouse infected with *P. yoelii* YM was graphed against the corresponding percentage of splenic PD1^+^CD4^+^ T cells **(B–D)** or the percentage of FOXP3^+^Tbet^+^ CD4 T cells **(F–H)**. Representative dot plots for PD1, FOXP3 and Tbet staining are shown in Supplementary Figure.

To determine whether improved survival after PD1 inhibition in the lethal malaria model resulted from altered T cell function or subsets, mice were infected with Py YM and then treated with LD01, α-PD1 mAb, or control rat Ig. Next, spleens were isolated at day 6 post-infection. PD1^hi^ T cells are considered to be markers for “exhausted” T cells during cancer. As such, we examined PD1^hi^ expression on CD4 T cells (Figure 5A), which are known to mediate survival from malaria (10). Interestingly, the number of splenic PD1^hi^CD4^+^ T cells significantly increased in mice treated with α-PD1 mAb relative to the control rat Ig while the LD01-treated mice remained similar (Figure 5A). A comparison of the percentages of PD1^+^CD4^+^ T cells vs. parasitemia showed significant correlations in all groups (Figures 5B–D). This association was strongest in the control rat Ig group (Figure 5B) with reduced correlation following α-PD1 mAb and LD01 treatment (Figures 5C,D). Of note, no correlation was seen between PD1^+^ expression on CD8 T cells and parasitemia (data not shown). The lethality of *P. yoelii* YM is multi-factorial (2, 7, 34, 35), and α-PD1 mAb or LD01 treatment may be modulating some of these factors to promote survival. Additional flow cytometry analysis demonstrated a significant decrease in the number of splenic FOXP3^+^Tbet^+^CD4^+^ regulatory T (Treg) cells (Figure 5E; Supplementary Figure), but not CD4^+^Tbet^+^FOXP3^−^ nor CD4^+^Tbet^−^FOXP3^−^ T cells (data not shown) in LD01 treated mice compared to control rat Ig. Moreover, when we compared the percentages of FOXP3^+^Tbet^+^CD4^+^ Tregs vs. parasitemia, we saw no correlation in the control rat Ig (Figure 5F) or α-PD1 mAb (Figure 5G); however, LD01-treated mice showed a significant correlation with low percentages of Tregs being associated with lower parasitemia (Figure 5H). Taken together, these data argue that LD01 treatment reduces the number of FOXP3^+^Tbet^+^CD4^+^ regulatory T cells resulting in the survival of mice infected with a lethal Plasmodium strain.

## Discussion

The data herein demonstrate that LD01, a short linear 22 amino acid peptide, antagonizes human PD1 as measured by an *in vitro* cell-based reporter assay. When dosed in combination with an adenovirus-based or irradiated sporozoite-based prophylactic malaria vaccine, LD01 enhances antigen-specific CD8+ T cell expansion supporting PD1 regulation of naïve-to-antigen-specific effector T cell transition and differentiation (34, 36, 37). Increased vaccine-induced CD8 T cell expansion following LD01 treatment suggests that the peptide is having an adjuvant-like effect, which corroborates work in non-human primates in which immunization with a SIVgag adenovirus-based vaccine in combination with α-PD1 mAb significantly elevated peak Gag-specific T cell responses (38). Additionally, LD01-enhanced T cell expansion in both immunization regimens indicates that its effects are independent of the vaccine platform. Studies are being planned to evaluate the efficacy of LD01 in combination with subunit, peptide-based, and nucleic acid-based vaccines. Moreover, because of the linear nature of the peptide, we are now attempting to express LD01 in several nucleic acid platforms including DNA and viral vectors. Further, since blockade of PD1/PDL1 and LAG-3/MHC II interactions have been shown to enhance T cell function and clearance of blood-stage parasites (7), future studies will also test the combination of LD01 and LAG3 inhibitors in both vaccine and therapeutic settings.

The enhanced CD8^+^ T cell expansion seen when LD01 is added to the adenovirus-based vaccine AdPyCS was associated with a modest increase in sterile protection following parasite challenge. Notably, the vaccine dose used in the challenge studies was sub-optimal and different from the dose tested when T cell expansion was assessed via ELISpot assay. Thus, future studies will assess the effect of LD01 on sterile protection with varying vaccine doses. In the RAS vaccine model, we were able to demonstrate that a single dose of LD01 at the time of immunization significantly enhanced the number of antigen-specific CD8^+^ T cells. Studies were not conducted to evaluate whether the increased antigen-specific CD8^+^ T cell expansion results in enhanced RAS vaccine efficacy; however, future studies are planned to assess this. The single dose of LD01 at the time of immunization argues that the timing of PD1 modulation during vaccination should be early during T cell priming. Nevertheless, we acknowledge that the dosing regimen for other vaccines may be different, thus we plan to preform studies to identify the optimal LD01 dosing regimen for current and future vaccine platforms we are testing. Because peptide-based biologics inherently have shorter pharmacokinetic half-lives, we would propose that LD01 acts in a pulsatile rather than sustained manner. In fact, pharmacokinetics analysis via LC/MS/MS in naïve mice treated IV with a single 200 μg dose of LD01 demonstrated that intact peptide circulated for <5 min, with metabolites of LD01 detected up to 120 min after administration (data not shown). Thus, we hypothesize that LD01 acts early during T cell proliferation and differentiation in a limited but sufficient therapeutic window, unlike the prolonged receptor blockade and overstimulation of the immune system by the mAbs. Moreover, these results provide support for the need to add a safe checkpoint inhibitor in combination with a T cell activating vaccine for infectious diseases, which is not unlike the therapeutic cancer vaccine strategy.

While a wide variety of liver-stage malaria vaccine strategies have induced strong CD8^+^ T cell responses and provided sterile immunity in healthy adults, these vaccines have been less effective in field evaluations in endemic regions (39, 40). Indeed, volunteers in malaria-endemic regions are often cleared of parasites before vaccine delivery by drug administration to eliminate the confounding of vaccine efficacy through the parasite's effects on the host's immune state (41). However, this approach does not take into account co-infection with other endemic pathogens such as helminths, *Mycobacterium tuberculosis*, and HIV, which have also been shown to suppress the host's immune responsiveness (4, 42). Further, it has been well-established that T cells can remain in an exhausted state upon termination of chronic antigen stimulation, and, in some cases, result in an epigenetic state of exhaustion (43–46). Therefore, it might not be sufficient to boost the CD8^+^ T cell responses alone with vaccines delivered in malaria-endemic regions. Rather, the exhausted immune system must be counteracted. In this regard, the delivery of LD01 or α-PD1 mAb to mice infected with lethal malaria protected approximately half of the infected animals. Minimal differences in survival were observed when LD01 treatment was initiated on day 1 relative to day 3 following infection suggesting that survival was not dependent on when LD01 was delivered. Notably, studies are being planned to determine an optimized therapeutic and prophylactic dosing strategy in the lethal malaria and vaccine models. Remarkably in the lethal malaria model, when the surviving animals were re-challenged at >160 days post-primary infection, all of the animals survived with no measurable parasitemia. We attribute this protective memory response seen in LD01 and anti-PD1 mAb-treated mice to enhanced memory T cell numbers and activity upon reinfection; however, further studies are required to determine whether this is the case and elucidate any role of protective antibodies. Taken together, these data suggest that including a safe checkpoint inhibitor as a therapeutic measure in combination with other anti-malarial drugs may increase efficacy and induce protective immunity. Indeed, when LD01 is delivered in combination with a vaccine, we would deliver a one-two punch in counteracting underlying immune exhaustion in endemic areas and simultaneously enhance effector T cell memory responses.

Intriguingly, we found that LD01 treatment of mice in the lethal malaria model significantly reduced the numbers of splenic FOXP3^+^Tbet^+^CD4^+^ Tregs compared to anti-PD1 mAb-treated mice. Moreover, anti-PD1 mAb significantly increased the numbers of splenic PD1^hi^CD4^+^ T cells relative to LD01-treated mice. Taken together, these data suggest a different mechanism of action between LD01 and the anti-PD1 mAb. In this regard, we have preliminary data showing that LD01 also specifically antagonizes the CTLA4 checkpoint receptor, supporting the notion that LD01 may contain polypharmacological properties. Moreover, the hydrophobic groove on PD1 that LD01 is computationally predicted to bind appears to be structurally conserved across a number of CD28 family receptors, including CTLA4. CTLA4 mAbs have been shown to enhance the anti-tumor immunity in humans by blocking activation of FOXP3^+^CD4^+^ Tregs (47). Thus, the reduction in FOXP3^+^Tbet^+^CD4^+^ Tregs with the potential polypharmacological properties of LD01 warrants further investigation, particularly in regards to potential mechanistic differences between LD01 and the mAb-based checkpoint inhibitors. This may have direct implications on improving the delivery of RAS malaria vaccine strategies that currently rely on IV delivery. When intradermal delivery of RAS was attempted, the vaccine failed to induce a response correlated with the malaria-specific Treg response that presumably blocked immunity to the parasite at the skin stage (48). Therefore, the delivery of the LD01 peptide transgenically from the parasite should also be explored.

In conclusion, the data indicate that our peptide-based immunomodulator, LD01, actively modulates the host adaptive immune system and is a viable modality for further development in combination with vaccines and as a therapeutic against chronic infectious disease agents.

## Data Availability Statement

All datasets presented in this study are included in the article/Supplementary Material.

## Ethics Statement

All studies and procedures were performed in accordance with strict institutional guidelines for animal care and use. Protocols were conducted in accordance with the National Institutes of Health guidelines and the Australian code of practice for the care and use of animals for scientific purposes (NHMRC, Australian). Protocol approvals were obtained from the QIMR Animal Ethics Committee (Approval Number A0209-622M) and the Institutional Animal Care and Use Committee of The Rockefeller University (Assurance No. A3081-01).

## Author Contributions

GG, VK, TP, JP, and MT contributed to the conceptualization and study design. Data organization and formal analyses were performed by GG, VK, TP, CB, AN, DK, MW, JH, and MT. Methodology and experiment executions were performed by JH, MT, MW, and DK. Supervision was contributed by GG, VK, TP, and JP. Writing of the original draft was completed by GG, VK, TP, CB, MW, and MT. JP, MM, AN, CB, MW, MT, PB, and DK performed manuscript review and editing. All authors contributed to the article and approved the submitted version.

## Conflict of Interest

TP, VK, CB, JP, AN, MM, PB, and GG were employed by the company Leidos, Inc. The remaining authors declare that the research was conducted in the absence of any commercial or financial relationships that could be construed as a potential conflict of interest.
