# Supplementary material for: A Peptide-Based PD1 Antagonist Enhances T-Cell Priming and Efficacy of a Prophylactic Malaria Vaccine and Promotes Survival in a Lethal Malaria Model
Source: Front Immunol. 2020 Jul 9;11:1377. doi: 10.3389/fimmu.2020.01377 (PMC7363839; doi:10.3389/fimmu.2020.01377)
Supplement: Supplementary file 1 [file Image_1.pdf]

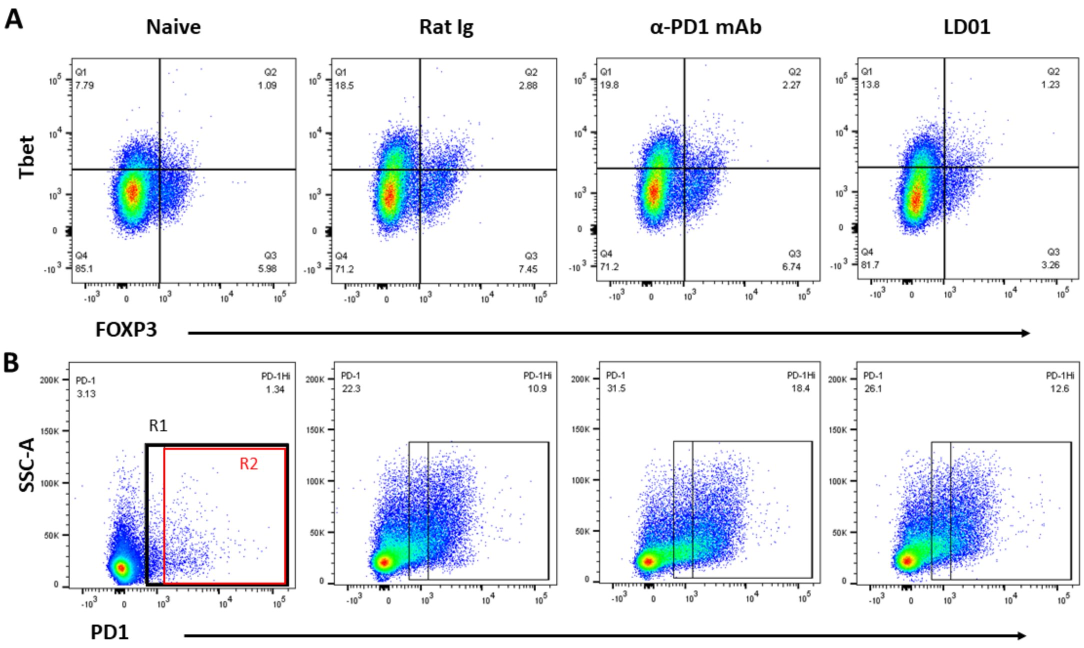

Supplementary Fig 1. Representative dot plots of PD1, FOXP3 and Tbet staining. Representative dot plots of Tbet and FOXP3 (A) or PD1 (B) expression on splenic-derived $CD3^+CD4^+$ T cells harvested at day 6 post-infection. In (B) the gated populations are as follows: R1 = $PD1^+CD4^+$ T cells; R2 = $PD1^{hi}CD4^+$ T cells.
